# The Performance of Short-Term Heart Rate Variability in the Detection of Congestive Heart Failure

**DOI:** 10.1155/2016/1675785

**Published:** 2016-11-06

**Authors:** Fausto Lucena, Allan Kardec Barros, Noboru Ohnishi

**Affiliations:** ^1^Universidade CEUMA, No. 100, 65903-093 Imperatriz, MA, Brazil; ^2^Laboratory for Biological Information Processing, Universidade Federal do Maranhão, S/N, São Luís, MA, Brazil; ^3^Graduate School of Information Science, Nagoya University, Furo-cho, Chikusa-ku, Nagoya-shi 464-8603, Japan

## Abstract

Congestive heart failure (CHF) is a cardiac disease associated with the decreasing capacity of the cardiac output. It has been shown that the CHF is the main cause of the cardiac death around the world. Some works proposed to discriminate CHF subjects from healthy subjects using either electrocardiogram (ECG) or heart rate variability (HRV) from long-term recordings. In this work, we propose an alternative framework to discriminate CHF from healthy subjects by using HRV short-term intervals based on 256 RR continuous samples. Our framework uses a matching pursuit algorithm based on Gabor functions. From the selected Gabor functions, we derived a set of features that are inputted into a hybrid framework which uses a genetic algorithm and *k*-nearest neighbour classifier to select a subset of features that has the best classification performance. The performance of the framework is analyzed using both Fantasia and CHF database from Physionet archives which are, respectively, composed of 40 healthy volunteers and 29 subjects. From a set of nonstandard 16 features, the proposed framework reaches an overall accuracy of 100% with five features. Our results suggest that the application of hybrid frameworks whose classifier algorithms are based on genetic algorithms has outperformed well-known classifier methods.

## 1. Introduction

Every year, congestive heart failure (CHF) related diseases are responsible for the death of millions of people around the world [1–3]. In this regard, large efforts are given to prolong the life of subjects [4]. Moreover, the treatment for cardiac pathologies is ranked amongst those with the highest cost for the healthcare system in low- and middle-income countries [1, 5]. Thus, Governments are enforcing the development of simple and low cost methods which can be able to detect heart failure on preventive exams. In fact, such an accomplishment would represent a breakthrough in the fight against life-threatening diseases [6].

At the clinical level, conventional methods to diagnose heart failure are based on a combination of tests (i.e., Valsalva maneuver, electrocardiography, echocardiography, and chest radiograph) and clinical history to determine whether or not the patient is afflicted with heart failure [7]. Among the tests used (i.e., Framingham, Duke, and Boston), the Boston criteria achieve sensitivity of 50% and specificity of 78%. Electrocardiography methods, such as electrocardiogram (ECG), through the analysis of abnormal ECGs reach sensitivity of 81.14% and specificity of 51.01% [8]. Echocardiograms show suboptimal values between 5% and 10% at rest and 20% and 30% under stress [9]. As one can see, the current problem of the conventional diagnose methods is the considerable difference between the percentages of correct and incorrect initial diagnoses [10]. A direct consequence is that false-negatives will cause unnecessary tests, whereas the false-positives will have late diagnostic. The diagnoses reliability, however, might be increased if the screening test of heart failure could be assisted by signal processing techniques and biomedical analysis. In the past years, several works [11–15] have shown the possibility of classifying subjects with heart failure. For instance, Işler and Kuntalp (2007) using short-term heart rate variability (HRV) intervals have shown that normalizing classical HRV and entropy measures can lead to high levels of sensitivity (82.76%) and specificity (100%). Kampouraki et al. (2009) suggested that the classification accuracy of heartbeat time series can be highly improved and even reach maximum accuracy if support vector machines (SVM) are used. A joint wavelet and SVM, for example, yield one of the highest success rates (98.61%) during the task of classifying CHF from normal sinus rhythm (NSR) [14]. Thuraisingham (2009) using second-order difference plot of RR intervals reported the best success rate (100%), but at the cost of long-term RR intervals (24 hours). There is also a wide range of studies that use multiscale entropy (MSE) as fundamental parameter as discriminative power [16]. As an example, a recent work has proposed the use of the reduced data dual-scale metrics in which the accuracy power has reached 100% using 500 RR samples (~10 minutes of ECG recordings) [17]. Yet, measures based on MSE are heavily biased on the number of samples, scales, and block analysis. A method based on classification and regression has shown a promising use of the short-term intervals. It demonstrates that sensitivity and specificity could reach, respectively, 89.7% and 100% by taking into account the average variation over 24 hours of consecutive heartbeat intervals [18]. Despite the number of sample tests and methodology used, the proposed techniques have different degrees of complexities. Specifically, they emphasize uncovering patterns that could be used to predict sudden death caused by heart failure. One interesting view of this problem is to find a representation that could be considered the representative pattern subserving the genesis of the autonomic cardiac control. In [19], for example, the authors show that it is possible to segregate cardiopathies by scaling the behavior of heartbeat intervals using wavelets.

Choosing what structures should be discarded or maintained during the analysis of ECG signals is a standard problem in clinical diagnosis. In this regard, one should comprehend the nature of the signal to infer the relevance of the structures composing its pattern. In this case, a common strategy to solve this problem has been to find patterns that are likely to appear when we are facing clinical alterations on subjects under observation. Usually a specialist needs to spend a longer time and effort analyzing data. Herein, we propose an alternative solution. A method that could help to predict congestive heart failure based on the analysis of short-term RR intervals (~5 minutes of ECG). With recent advances on computer-aided detection and diagnosis systems, the need of simple and accurate methods plays an important role, especially in telemedicine. The novelty described here shows the capacity of indicating the presence or absence of a cardiac disease. Yet, our methodology can be extended to other areas, such as detection of breast cancer [20], diabetes [21], and even distinguishing different modalities of motor imagery based on EEGs analysis [22]. Last but not least, our idea is also patients in remote areas, that is, where one does not have easy access to diagnosing tools. For instance, there are areas where there is only an ECG available and usually no specialist, but a general clinician (a problem that we currently see in some rather poorer regions in Brazil) [6].

This paper is described in the following sections, where Section 2 covers the matching pursuit algorithm.  Section 3 describes the database used. Sections 4 and 5, respectively, explain the feature extraction and feature subset selection. The overview of the system is given in Section 6. At last, discussion, results, and conclusions can be found from Sections 7 to 9.

## 2. The Matching Pursuit Algorithm

Several models of autonomic cardiac regulation are based either on the analysis of input-output relationship [23–25] or on the idea of selective frequency extraction [26]. Altogether, they often explore the standard frequency division suggested to analyze the HRV signals [27]. A simple way to accomplish this task is to use the Fourier transform or autoregressive methods (AR). A drawback, however, is that Fourier and AR methods are not robust to nonstationarity. An alternative way has been to use time and frequency transformations to overcome nonstationarity. Essentially, one can drop the nonstationarity problem by selecting a function that decomposes a signal into a sequence of bases using adaptive time-frequency transform (ATFT) algorithms. This approach is accomplished by scaling, translating, and modulating versions of the basis function, such that they represent the decomposed signal with a well-defined time and frequency distribution. For instance, ATFT algorithms have drawn a lot of attention in pattern classification [28] and signal compression due to their capacity of reducing a higher dimension space to a few numbers of parameters. One of the most used ATFT algorithms exploits a matching pursuit (MP) decomposition [29, 30]. The MP framework represents a signal *x*(*t*) as a linear combination of *N* basis functions *ϕ*(*t*) drawn from an overcomplete dictionary Φ = [*ϕ*_1_,…, *ϕ*_*M*_], where *M* ≫ *N*, or alternatively

(1) $$\begin{array}{c} x\left(\;t\;\right)\approx \overset{N}{\underset{n=1}{\sum }}c_{n}\phi_{n}\left(\;t\;\right) \end{array}$$

in which *ϕ*(*t*) can be Gabor functions described as

(2) $$\begin{array}{c} \phi \left(\;t\;\right)=Ae^{-\pi {\left(\left(t-u\right)/s\right)}^{2}}\cos\left(\;w\left(\;t-u\;\right)+\varphi \;\right), \end{array}$$

where *c*_*n*_ means modulatory coefficient, *s* is scale, *w* is frequency modulation, *u* is translation, *φ* is phase, and *A* is a normalization factor, such that ‖*ϕ*(*t*)‖ = 1. Based on previous studies [31], we know that the structures underlying the heartbeat intervals components have a Gabor-like representation. Using the MP based on the decomposition of the heartbeat intervals by Gabor functions, it is possible capture representations in terms of coherent and noncoherent structures [32]. In one hand, coherent structures can be understood as the Gabor functions (which compose the dictionary) that have the highest correlation with the decomposed interval. On the other hand, noncoherent structures are likely to represent noise-like random structures which are not well defined in terms of time and frequency representation. They are likely to have small correlation with the decomposed interval.

The MP decomposes *x*(*t*) by finding the best orthogonal projections amongst a set of basis functions from a dictionary Φ that matches the structure of *x*(*t*). It results in a finite number of basis functions organized in decreasing order of energy.

**Figure 1:**
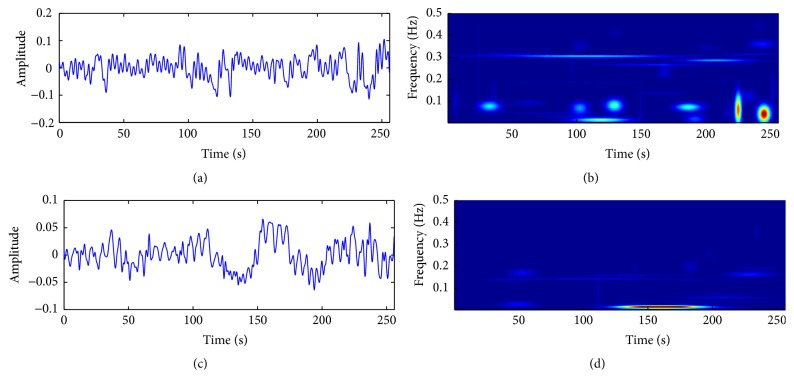
Example. Heart rate variability (zero mean) and its joint time and frequency domain. Each “circle” (b and d) represents a Gabor function chosen by the MP algorithm. Normal sinus rhythm (a and b) and congestive heart failure signal (c and d). Both time-frequency planes are normalized to have the same energy levels (scale omitted for better visualization).

A fundamental aspect of MP algorithm is how the signal is decomposed [32]. That is, because not all the signals are composed of well-defined (coherent) components, the MP tends to decompose coherent underlying structures first and then break random spike-like noise structures into a set of basis functions whose time and frequency distribution are less compact than coherent ones.  Figure 1 illustrates an example of MP decomposition using CHF and NSR HRV waveforms followed by their time-frequency representation. It shows remarkable differences between time and frequency plane. Such differences are likely to be associated with the temporal variations of HRV intervals.

## 3. The Dataset

We applied the MP algorithm to intervals containing 1024 HRV continuous samples randomly obtained from CHF patients and NSR volunteers of two well-known datasets (we have used 256 RR continuous samples to emulate the short-term analysis of ECG waveforms). This process is followed by resampling the unevenly RR intervals at 4 Hz (resulting in 1024 samples evenly distributed in time) and removing the linear trend of the HRV signal. Herein spline-cubic interpolation was used as resampling method and the detrending approach was performed using smoothness priors, similarly to a time-varying FIR high-pass filter [33]. The CHF dataset (http://physionet.org/physiobank/database/chf2db/) is composed of 29 ECG long-recording signals (24 hours) acquired from patients without any control protocol, whose age ranges from 34 to 79 years. CHF is basically classified by the New York Heart Association [34] into four different classes, each one expressing how the CHF is evolved in terms of physical activity. In class I, there are neither evident symptoms nor limitations of any kind of physical maneuvers, and the subjects are able to perform simple day-life activities. In class II, the subjects start to have mild indicators of a cardiac disease, such as small resistance to physical activity and difficulty in breathing. In class III, the symptoms are worse; there are notable physical limitations. The subjects are unable to do less-than-simple physical activities without pain, for example, walking long distances or climbing stairs. In class IV, the subjects are incapable of performing any kinds of activities and feel pain even in inactive states. These are bedridden patients. Herein the database is composed of subjects selected from NYHA classes I, II, and III. The gender of 10 patients is specified (eight men and two women), but unknown for the remaining. The NSR dataset (http://physionet.org/physiobank/database/fantasia/) is used as a control group. It is composed of 40 ECG waveforms (two hours) recorded from healthy volunteers during supine resting while watching the movie Fantasia (Disney, 1940). This dataset was divided into two groups: young (21–34 years old) and elderly (68–85 years old). Each group contains the same number of men and women. Both CHF and NSR datasets were, receptively, digitalized at 128 Hz and 250 Hz. The beats from each ECG were carefully cataloged through unsupervised systems followed by visual inspection of experts.

## 4. Heartbeat Intervals Feature Extraction

### 4.1. Mean Energy Decay Rate

**Figure 2:**
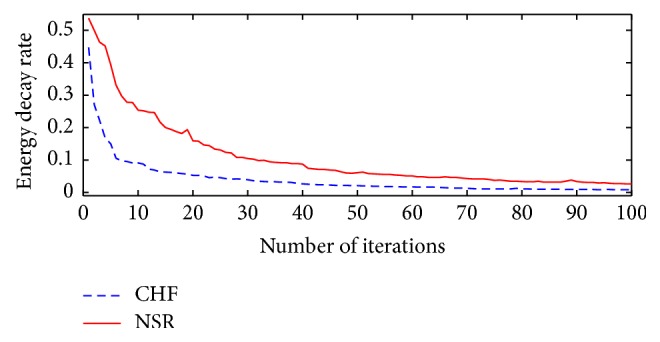
Energy decay ratio. The residual energy after each MP iteration for CHF (dash line) and NSR (solid line). Slow decays suggest that the decomposition was carried out by noncoherent structures (NSR) in opposition to coherent structures (CHF).

In Section 2, we have explained that the MP algorithm works by selecting a basis function by projecting it onto an analyzed signal, such that it captures the maximum amount of energy of the signal through the basis function. According to the structure of the signal, the MP algorithm can decompose the signal using few basis functions, if it is composed of coherent structures. On the other hand, noncoherent structures are likely to require a higher number of MP iterations. It can be noted that the nature of the decompositions (based on coherent and noncoherent structures) alters the residual energy decay that varies from signal-to-signal [32, 35, 36]. Comparing CHF and NSR energy decay rate, it is possible to observe (Figure 2) that CHF has a faster decay when compared to NSR. Based on this observation, one can use the mean energy decay as a feature to differentiate between NSR and CHF. Thus, we define the mean energy decay rate as the average of the residual energy, which is derived from the difference between the signal being analyzed and its reconstructed version at each iteration. We express the residual energy rate in function of the iteration number *m* as

(3) $$\begin{array}{c} E_{r}^{m}\left(\;t,w\;\right)=E_{x}\left(\;t,w\;\right)-\overset{m}{\underset{n=1}{\sum }}{\left|\;c_{n}\;\right|}^{2}W_{\phi_{n}}\left(\;t,w\;\right), \end{array}$$

where *𝒲*(*t*, *w*) is the Wigner-Ville distribution [29].

The averaged measure of *E*_*r*_^*m*^(*t*, *w*) gives the mean energy decay rate and it is then computed as

(4) $$\begin{array}{c} E\left(\;t,w\;\right)=\frac{1}{M}\overset{M}{\underset{m=1}{\sum }}E_{r}^{m}\left(\;t,w\;\right), \end{array}$$

where we calculate *E*(*t*, *w*) for each (very-low frequency) VLF, (low frequency) LF, and (high frequency) HF band.

### 4.2. Features Based on the Power Spectrum Density

A standard measure to analyze the reciprocal relationship between the autonomic branches (SNS and PNS) is the ratio between the LF and HF bands [27]. This ratio has been often used to show the degree of the modulatory mechanisms acting into the heart [37]. It has been reported, however, that patients with CHF have a remarkable reduction of energy at HF bands following a high increase of energy at VLF bands [38]. Therefore, one may expect that dividing the energy at HF by the VLF band causes an enhancement onto this ratio, such that the ratio value for CHF tends to be lower than NSR (see Figure 5). The frequency ratio can be obtained by dividing the power spectrum density of HF by the VLF. Herein, we combine the *ϕ*(*t*) whose center frequencies are located at VLF, LF, and HF to construct subsignals and thus obtain their PSD [39, 40]. The PSD is computed through Welch's periodogram using the* pwelch* function (MATLAB environment program). Briefly, let us denote a linear combination of *ϕ*(*t*) as *x*(*t*), where *x*(*t*) is divided into *K* frames of size *M* whose *m*th windowed, zero-padded frame with successive *R* samples and rectangular window *h*(*n*) are

(5) $$\begin{array}{c} x_{m}\left(\;n\;\right)=h\left(\;n\;\right)x\left(\;n+mM\;\right),\;m=0,1,\ldots ,K-1 \end{array}$$

with

(6) $$\begin{array}{c} h\left(\;n\;\right)=\left\{\begin{array}{cc} 1 & if\;\;n=0,\ldots ,M-1\\ x & if\;\;otherwise, \end{array}\right. \end{array}$$

and *n* = 0,1,…, *M* − 1, where the periodogram of *m*th block is expressed as

(7) $$\begin{array}{c} P_{x_{m},M}\left(\;w_{k}\;\right)=\frac{1}{M}{\left|\;\sum_{n=0}^{M-1}x_{m}\left(\;n\;\right)e^{-j2\pi nk/M}\;\right|}^{2}, \end{array}$$

where *w*_*k*_≜2*πk*/*M* and *k* = 0,…, *M*/2.

Using (7), the power spectrum density using Welch's method is then represented as [41]

(8) $$\begin{array}{c} {\hat{S}}_{x}\left(\;w_{k}\;\right)=\frac{1}{K}\overset{K-1}{\underset{m=0}{\sum }}P_{x_{m},M}\left(\;w_{k}\;\right). \end{array}$$

The HF/VLF ratio and PSD of LF are defined as

(9) HFVLF=∑wk∈HFS^xwk∑wk∈VLFS^xwk,LF=∑wk∈LFS^xwk.

### 4.3. Entropy Based on MP Decomposition

According to the MP decomposition, any signal *x*(*t*) can be decomposed as a linear combination of *N* basis functions and weight coefficients. From (1),

(10) $$\begin{array}{c} x\left(\;t\;\right)\approx c_{1}\phi_{1}\left(\;t\;\right)+c_{2}\phi_{2}\left(\;t\;\right)+c_{3}\phi_{3}\left(\;t\;\right)+\cdots +c_{N}\phi_{N}\left(\;t\;\right), \end{array}$$

where the energy of each component *c*_*n*_ *ϕ*_*n*_(*t*) is represented by *E*_*n*_ = |*c*_*n*_ *ϕ*_*n*_(*t*)|^2^ with total energy *E*_*x*_ = ∑_*n*_ *E*_*n*_. If the dictionary is complete, then the probability distribution of *x*(*t*) can then be seen as the sum of individual probability contributions given by each component as

(11) $$\begin{array}{c} \overset{N}{\underset{n=1}{\sum }}p_{n}\left(\;x\;\right)=1, \end{array}$$

where *p*_*n*_(*x*) = *E*_*n*_/*E*_*x*_. Using the definition of entropy given by Thomas [42], the entropy *H*_*w*_ of the probability distribution defined in (11) is calculated as

(12) $$\begin{array}{c} H_{w}\left(\;p\;\right)=-\overset{N}{\underset{n=1}{\sum }}p_{n}{log}_{2}\left(\;p_{n}\;\right). \end{array}$$

In this paper, *H*_*w*_(LF) and *H*_*w*_(HF) correspond to the entropy of the components whose frequencies belong to LF and HF bands, respectively.

### 4.4. Central Frequency Distribution

**Figure 3:**
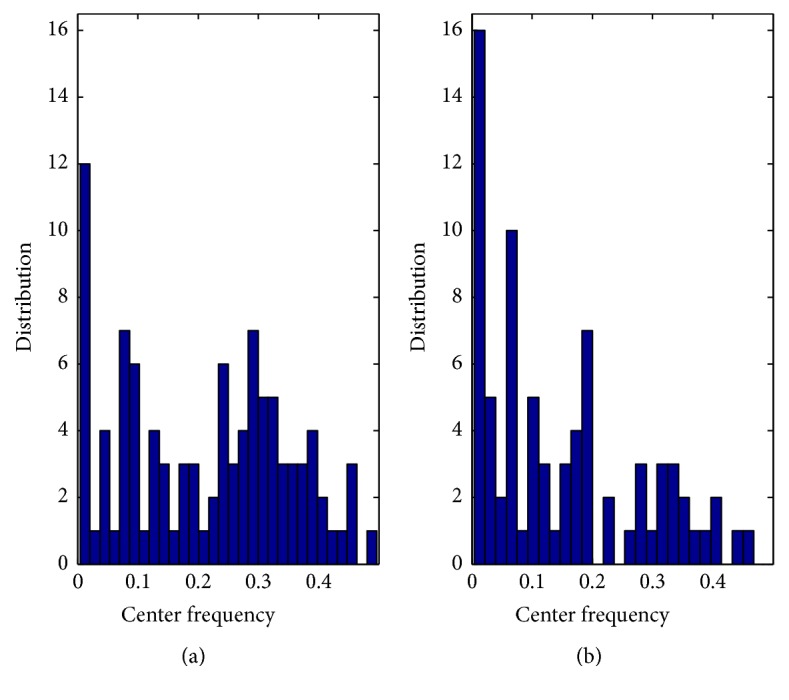
Example of the frequency distribution measurement pattern. Number of Gabor functions (distribution) organized by their center frequency (Hz). (a) Normal sinus rhythm. (b) Congestive heart failure.

It is clear from the time and frequency plane, shown in Figure 1, that there are remarkable differences on the energy signature given by the frequency distribution of each basis function for CHF and NSR. Therefore, the frequencies [*w* in (2)], which were obtained from the structures that decompose the HRV signal, may be used to reflect the frequency distribution of the basis functions according to the HRV frequency band division (i.e., VLF, LF, and HF). To capture these patterns, we use a feature based on the frequency distribution *D* represented by [32]

(13) $$\begin{array}{c} D\left(\;w\;\right)=\frac{M_{cf}\left(w\right)}{M}, \end{array}$$

where *M*_cf_ accounts for the number of basis functions whose central frequency is on either VLF, LF, or HF bands. Moreover, *M* represents the total number of basis functions (which were constraint to 30) that are used to reconstruct the original signal.  Figure 3 shows an example of the frequency population for NSR and CHF. It illustrates how the dynamical behavior of the HRV is captured by the MP algorithm in relation to the frequency distribution of the basis functions. It is evident that there is a decrease of frequency distribution in HF bands and an increase between VLF and LF bands in CHF when compared with NSR volunteers. Since our goal is to capture the variations of the frequency population (for using them as discriminative patterns between CHF and NSR), we applied the frequency distribution to VLF, LF, HF, HF/VLF, and VLF/LF bands. Note, however, that if an elevate number of basis functions are concentrated at a certain center frequency (Figure 3), it does not mean energy concentration. But, a higher number of structures are necessary to approximate the original signal by means of basis functions with low energy concentration.

## 5. Feature Subset Selection

Feature subset selection (FSS) is a process that deals with the problem of identifying quasi-optimal combination of patterns-representing features among a large set of features. In pattern classification problems, FSS has been used to improve the overall accuracy of the classifier. It can be considered a special case of feature selection where a weight value is assigned to each feature using binary strings. FSS is basically divided into filter or wrapper based-approaches. That is, if there is dependency between the classifier and the learning algorithm, FSS falls under the rubric of the* filter* approach; otherwise, it is called* wrapper*. In this work, we use a* filter* approach based on genetic algorithms to select the most suitable subset of features to detect CHF from a control group composed of NSR volunteers.

### 5.1. The Learning Algorithm

In the proposed system, we use a genetic algorithm (GA) as learning algorithm. In brief, GAs use principles derived from natural selection and genetics to perform randomized search in complex landscapes. They have been largely used to provide quasi-optimal solutions in optimization problems, such as pattern recognition and machine learning [43]. In GA, a binary population representing a space of feature subsets is constructed based on structures called chromosomes, where each *i*-element of the binary chromosome string *c*_*n*_  {*c*_1_, *c*_2_,…, *c*_*n*_} is correlated with the absence {*c*_*i*_ = 0} or presence {*c*_*i*_ = 1} of a feature. For instance, a chromosome represented by “100100000” means that features *c*_1_ and *c*_4_ were selected to construct a classifier.

### 5.2. The KNN Classifier and Feature Scaling

A supervised classification system based on the *k*-nearest-neighbor (KNN) rule describes a method where a set of *N* labeled pattern vectors **s**_1_, **s**_2_,…, **s**_*N*_ (previously assigned to one of the *M* classes *C*_1_, *C*_2_,…, *C*_*M*_) is used to determine to which class *C*_*i*_ a new feature vector **x** belongs, according to the following rule [44]:

(14) $$\begin{array}{c} D\left(\;s_{i},x\;\right)=\min\left\{\;D\left(\;s_{i},x\;\right)\;\right\}\;if\;\;x\in C_{i}, \end{array}$$

where *i* = 1,2,…, *N* and *D* represents the Euclidean distance metric between two feature vectors as *D*_*i*_^2^ = ‖**x** − **s**_*i*_‖^2^ = ∑_*i*=1_^*n*^(*x*_*i*_ − *s*_*i*_)^2^ [45].

The classifier discrimination power can be increased if a feature value scaling is used to reduce great numeric ranges among the feature vectors [11]. A procedure, known as MinMax, where the feature vector **x** is scaled between 0 and 1, is expressed as

(15) $$\begin{array}{c} x^{\prime }=\frac{x-\min\left\{x\right\}}{\max\left\{x\right\}-\min\left\{x\right\}}, \end{array}$$

where **x**′ is the normalized feature vector.

### 5.3. Validation and Performance Assessment

#### 5.3.1. The *k*-Fold Validation

To validate the system, the feature dataset composed of *n* samples is normally divided into a test and a training set. The purpose of a training set is to regulate the parameters of the classifier according to the input examples, while the test set yields the overall accuracy of the system. A drawback, however, is that a biased estimator of the discriminative performance can occur if repeated samples are occasionally tested. A faithful way of estimating the system performance is to use a *k*-fold cross-validation [46]. In this cross-validation version, the dataset is segregated into *k* subsets (almost) of equal size, where *k* − 1 subsets are used to train and the remaining subset is used as testing set. This process is repeated until all the folds are tested and their results averaged. Because the test set is disjoint of the training samples and used just once, the independence between training and test sets is maintained. It should be pointed out that the standard deviation for sensitivity, specificity, and accuracy increases as the number of folds is reduced. Thus, there is a trade-off between the number of folds and the performance of the cross-validation method. Therefore, choosing a high value for the number of folds ensures a low variance for performance evaluation since we can assume that any classifier has bias effects [47].

Herein the dataset is composed of 69 samples and they were divided into 23 folds, where 66 samples are used as training set and three samples as test per fold time. The averaged results of the test set are then used to evaluate the fitness value *θ*, which tries to minimize the error rate of the classifier according to

(16) $$\begin{array}{c} \theta =1-\frac{Number\;\;of\;\;samples\;\;correctly\;\;classified}{Total\;\;number\;\;of\;\;samples}. \end{array}$$

#### 5.3.2. Performance Measures

Performance measures are results-based decisions traditionally organized into a confusion matrix. This matrix describes if the samples assigned by the classifier to the presence (true) or absence (false) of the disease are in fact correct (positive) or incorrect decisions (false). The three most common performance measures are sensitivity [Se = TP/(TP + FP)], specificity [Sp = TN/(TN + FP)], and accuracy [Ac = (TP + TN)/(TP + TN + FP + FN)], where TP, TN, FP, and FN correspond, respectively, to true positive, true negative, false positive, and false negative. Se, Sp, and Ac are, in this order, connected to the indicative presence or absence of illness and general performance of the classifier.

## 6. System Overview: Implementation Details

**Figure 4:**
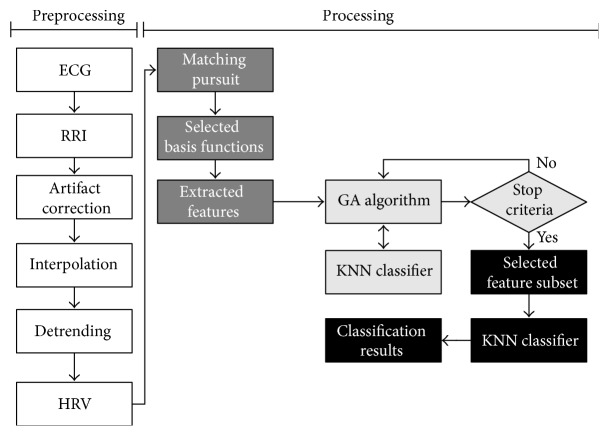
System overview flowchart. Preprocessing stage (*white boxes*). Processing stage: matching pursuit decomposition and feature extraction (*dark gray boxes*), KNN/GA algorithm optimization (*light gray boxes*), and final classification results based on KNN classifier (*black boxes*).

An overall view of the system is illustrated using flowchart diagram in Figure 4. The system is basically divided into two stages—preprocessing and processing—where the second stage is composed of three steps:

1. Feature extraction based on matching pursuit algorithm.
2. Feature subset selection using the KNN/GA algorithm.
3. Overall classification.

In the* first step* of processing, the resulting HRV signal is decomposed using the MP algorithm and its reconstructed signal obtained using 30 basis functions. Using the decomposed basis functions 16 features were extracted,* namely*, residual energy {*E*(VLF), *E*(LF), *E*(HF), *E*}, PSD based energy concentration {VLF, LF, HF, HF/VLF, HF + LF}, entropy {*H*_*w*_(LF) and *H*_*w*_(HF)}, and frequency distribution {*D*(LF), *D*(HF), *D*(HF)/*D*(VLF), *D*(VLF)/*D*(LF), *D*(LF)/*D*(HF)}. In the* second step*, we used the combined KNN classifier and GA algorithm to simultaneous model optimization for feature subset selection based on the Bioinformatics and Genetic Algorithm MATLAB Toolboxes (The Mathworks, 2007). In brief, it runs a standard genetic algorithm in which the selection uses a rank-based strategy where the two highest ranked chromosomes are selected to survive to the following generation. The feature subset selection results are based on a 23-fold cross-validation method whose parameters setting for the binary population size is 300 and the number of generations is 100, with crossover probability (*P*_*c*_) of 0.7 with a double string crossover and mutation probability (*P*_*m*_) of 0.05.

Once the stop criteria are reached—either by succeeding the number of generations or when the fitness value does not decrease in the last 30 generations—the joint KNN/GA optimization algorithm yields the best selected feature subset, that is, the feature subset whose discriminative power has one of the lowest error rates to discriminate CHF from NSR. The* third step* consists of using the selected feature subset to validate the performance of the yielded features.

## 7. Results

We have tested the discriminative power of the features derived from the MP decomposition with and without a strategy to select the best feature subset. We also investigated if scaling the features, which overcome exaggerated discrepancies among the numeric values, could improve the overall classification rate.  Table 1 shows the results, namely, accuracy, sensitivity, specificity, and number of features (used or selected).  Table 1 is divided into different configurations where the used *k*-nearest neighbors in the classifier are 1, 3, 5, 7, 9, 11, and 13. The configurations are organized in (a) KNN classifier using all (16) features with feature scaling, (b) KNN classifier using all (16) features without feature scaling, (c) FSS based on KNN/GA algorithm with feature scaling, and (d) FSS based on KNN/GA algorithm without feature scaling.

**Figure 5:**
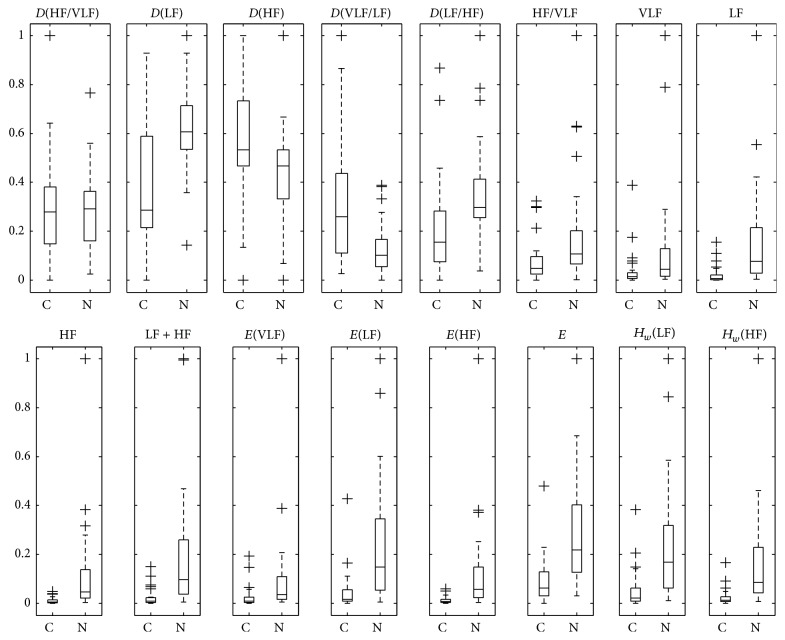
Box plot of features computed for NSR and CHF heartbeat intervals. The central mark represents the median for 40 volunteers (NSR) and 29 subjects (CHF); the edges of the box are the 25th and 75th percentiles. While outliers are plotted individually (+), the whiskers are considered not outliers.

In configuration (a), the highest accuracy (95.65%) was obtained with *k* = {3}, followed closely by *k* = {1,5, 7,9, 11,13}, whose accuracy is 92.75%. Configuration (b) yielded a lower accuracy rate (94.20%) than (a). Configurations (c-d) show a substantial improvement of system accuracy. Specifically, when compared to configuration (a-b), the system improvement ranges from 4.35% to 26.09%. For instance, the best accuracy is obtained in configuration (c), where the system reached its maximum performance (Ac = Se = Sp = 100%) using only five features. The selected features for *k* = {5} are {*D*(HF)/*D*(VLF), *D*(LF), VLF, *E*, *H*_*w*_(HF)}. We show the numeric values of the computed features to CHF and NSR after MinMax scaling in Figure 5. In spite of their overlapping ranges, frequency distribution *D*(·) feature was selected as being a “good” discriminant between NSR and CHF. Analysis of the individual features shows that *D*(HF)/*D*(VLF) was spanned over 0.32 ± 0.23 (mean ± SD) for CHF. The NSR, however, was spread in a much lower range (0.28 ± 0.15). At first sight, *D*(LF) seems to have a high discriminative power. In fact, their values are distributed over 0.38 ± 0.24 for CHF against 0.61 ± 0.17 for NSR.

It has been also reported that energy-based measures derived from HRV signals are strong discriminant features between NSR and CHF. In our case, VLF and LF + HF were selected as subset features. In one hand, VLF has values at 0.10 ± 0.19 for NSR and 0.03 ± 0.07 for CHF. On the other hand, LF + HF has values at 0.18 ± 0.19 (NSR) and 0.02 ± 0.07 (CHF).

Another selected feature was the residual energy decay rate (*E*), which is strongly dependent on the MP algorithm decomposition. Their values are 0.08 ± 0.09 (CHF) and 0.27 ± 0.19 (NSR). Nevertheless, the last feature selected by the joint KNN/GA algorithm is the entropy based on MP decomposition for HF bands with 0.15 ± 0.18 (NSR) and 0.02 ± 0.03 (CHF).

## 8. Discussion

There is a great number of works dealing with the problem of discriminating CHF from NSR. Despite the used techniques, they can be divided into analysis applying long-term or short-time intervals of HRV signals. Their goal is to extract features whose discriminatory power could help to identify pathological characteristics. It is evident, however, that long-term recordings underlie a higher degree of regulatory information than short-term intervals. Consequently, they are largely preferred by the majority of studies in the task of classifying CHF from a given group. The problem of using long-term intervals is that it requires a continuous monitoring of the cardiac activity during long hours. Short-term intervals, on the contrary, can be advantageous if the first symptoms of CHF can be identified in a short interval of time. Herein we focus on a discriminative method for CHF under the rubric of short-term intervals.

One of the claimed challenges in discriminating CHF from NSR using short-term intervals is that five minutes (or less) may not be enough to fully characterize the day-life activity of the heart. We have shown that, using an adaptive decomposition based on the MP algorithm, one can analyze the basis functions used to decompose the signal instead of the HRV signal itself. The novelty of this analysis lies in using the underlying structural complexities of NSR and CHF as discriminatory basis. That is, NSR requires a higher number of noncoherent structures than CHF to be decomposed, which causes a slower decay of energy (*E*). Moreover, each basis function corresponds to a specific position on the time and frequency plane (see Figure 1). Their frequencies distribution (*D*) carries important information about the decomposed signal (see Figure 3). We have also introduced a flexible way of measuring information from the HRV signals. Computing entropy (*H*_*w*_) based on the MP algorithm allows one to estimate entropy directly from the decomposed basis functions [48]. This method represents a much more flexible way to estimate entropy from the standard frequency division (VLF, LF, and HF) than using multiresolution decomposition [49].

Our method was able to predict CHF in patients using short-term HRV intervals. However, it does not indicate which functional capability (classes NHYA I to IV) neither the objective assessment (classes A to D) of each patient under analysis [50]. Previous studies suggest that the heartbeat intervals are highly sparse [31]. Therefore, a possible solution to solve this problem is adding a feature based on high-order statistics that is sensitive to small variations on sparse data.

In the MP decomposition, the largest energy Gabor components that compose the signal are extracted first, while they are mostly located at higher frequencies. Gradually, the signal continues to be broken into lower energy components. Thus, the components located at very-low frequency approache zero energy due to their very slow fluctuations. This property is captured by the central frequency distribution, as shown in Figure 3. Therefore, our analysis is likely to be less sensitive to the effect of the trend contribution.

Regarding the analysis, it is important to notice that there are differences between MP and traditional Fourier-based methods, such as the periodogram. The periodogram, which is given by the modulus squared of the discrete Fourier transform, is not an efficient estimator. That is, it does not converge to the true spectral density due to the finite length of the method window-based analysis. Therefore, the periodogram is not robust to background noise during the analysis of instantaneous signals, such as HRV. It has been reported, however, that MP algorithms have a higher performance to detect instantaneous signals (such as evoked potentials and HRV signals) even under the effect of heavy background noise [51].

Another relevant problem, which is related to feature selection, was circumvented by using a hybrid architecture (KNN/GA). In this regard, we have shown that configuration (c) with *k* = 5 has the lowest error rate and one of the minor numbers of features among the other configurations. The selected features by the KNN/GA algorithm yield a subset selection containing five features with high discriminative power. According to Figure 5 and mean ± SD of the features, one may organize selected features in decreasing order of discriminative power as *H*_*w*_(HF), *E*, LF + HF, VLF, *D*(LF), and *D*(HF)/*D*(VLF). But, it should be noticed that the classification results may vary according to the number of *k*-nearest-neighbors used or different classifier methods.

One argument to explain the different results among the configurations with and without MinMax procedure is based on the classifier (see Table 1). That is, the KNN classifier tends to assign the test sample to the class according to the Euclidean distance. Therefore, if the feature space is composed of a large number of features with considerable numerical variations among them, then the KNN classifier will have a high probability of assigning the test sample to a wrong class. The KNN classifier rule, however, tends to increase the classification accuracy when the feature space has their numerical variations reduced. This property of the KNN classifier is not noticed during the KNN/GA optimization, because this procedure selects the features whose output is based on increasing the accuracy of the system.

## 9. Conclusion

As conclusion remarks, this work shows that using short-term intervals based on MP decomposition it is possible to discriminate CHF from NSR with low error rate. Unlike what someone may suggest, only few features are necessary to carry out this task. Our work holds interesting advantages in comparison to the previous studies on the same subject. In special, because it can be extended to discriminate not only CHF, but also other cardiac pathologies in which similar patterns can be further applied to short or long intervals of HRV. We believe that successful application of the discriminant analysis in cardiology (such as the one described here) can represent an important tool to the clinician in areas where healthcare is less feasible (i.e., remote communities) through telemedicine.

## Figures and Tables

**Table 1:** Classification results. KNN (*k*-nearest-neighbor) with and without MinMax normalization. KNN/GA (genetic algorithm) optimization with and without MinMax normalization. The results use a 23-fold cross-validation where Ac (accuracy), Se (sensitivity), and Sp (specificity) quantify the performance assessment of the classifier using *N* features.

Algorithm method	With MinMax normalization					Without MinMax normalization			
	*k*	Ac (%)	Se (%)	Sp (%)	Features	Ac (%)	Se (%)	Sp (%)	Features
KNN	01	92.75	78.26	100.0	16	73.91	78.26	71.73	16
KNN	03	95.65	86.95	100.0	16	82.60	60.87	93.47	16
KNN	05	92.75	78.26	100.0	16	89.85	82.60	93.47	16
KNN	07	92.75	78.26	100.0	16	86.97	73.91	93.47	16
KNN	09	92.75	78.26	100.0	16	91.30	82.60	95.65	16
KNN	11	92.75	78.26	100.0	16	92.75	82.60	97.82	16
KNN	13	92.75	78.26	100.0	16	94.20	86.95	97.82	16

KNN/GA	01	98.55	100.0	97.82	09	92.75	91.30	93.47	03
KNN/GA	03	98.55	95.65	100.0	08	94.20	95.65	93.47	03
KNN/GA	05	**100.0**	**100.0**	**100.0**	**05**	94.20	91.30	95.65	08
KNN/GA	07	98.55	95.65	100.0	05	95.65	91.30	97.82	07
KNN/GA	09	98.55	95.65	100.0	05	94.20	91.30	95.65	07
KNN/GA	11	98.55	95.65	100.0	05	94.20	86.95	97.82	09
KNN/GA	13	98.55	95.65	100.0	06	94.20	86.95	97.82	07
